# Aging Redefined: Cognitive and Physical Improvement with Positive Age Beliefs

**DOI:** 10.3390/geriatrics11020028

**Published:** 2026-03-04

**Authors:** Becca R. Levy, Martin D. Slade

**Affiliations:** 1 Social and Behavioral Sciences Department, Yale School of Public Health, New Haven, CT 06520, USA; 2 Psychology Department, Yale University, New Haven, CT 06520, USA; 3 Department of Internal Medicine, Yale School of Medicine, New Haven, CT 06520, USA; martin.slade@yale.edu

**Keywords:** aging, cognitive health, mental health, improvement, age beliefs, trajectories

## Abstract

**Background/Objectives**: A widespread assumption exists among scientists, health care providers, and the public that later life is a time of inevitable and universal cognitive and physical decline. This assumption is likely due to considering older persons who improve to be exceptions, and the reliance on aging-health measures that do not allow for improvement. In contrast, we utilized a measure that allowed for an upward trajectory to occur. Our objective was to examine whether a meaningful number of older persons improve with this measure and, if so, to examine whether a promising modifiable culture-based variable, positive age beliefs, contributes to this improvement. **Methods**: Individuals 65 years and older, who participated in a nationally representative longitudinal study, had their physical health assessed by walking speed and their cognitive health assessed by a global performance measure. We calculated the percentage of the sample that showed improvement in each domain from baseline to the last measurement up to 12 years later. We also examined whether a positive-age-belief measure predicted this improvement in regression models. **Results**: It was found that 45.15% of persons improved in cognitive and/or physical function over this period, and positive age beliefs predicted these two types of improvement, both with and without adjusting for relevant covariates. **Conclusions**: Our findings underscore the need to instill or magnify the positivity of age beliefs and to redefine aging so that it includes the possibility of improvement.

## 1. Introduction

In the last stage of a long career, Joseph Turner created his most innovative and influential paintings; Diana Nyad set a world record in her 110-mile swim from Cuba to Florida at the age of 64, after several attempts while younger. These disparate accomplishments demonstrate that improvement can occur in later life. A goal of the current study was to examine whether later-life improvement in cognitive and physical functioning not only occurs among extraordinary older persons but also among those in the older general population.

If that improvement is found, it would contradict a dominant belief about aging held by scientists, health care professionals, and the lay public that it is a time of inevitable and universal decline in functioning. A review of scientific definitions concluded that “Aging is consensually described as a process of loss” [1]. A global survey of nearly 40,000 people found that 65% of health care professionals and 80% of lay persons falsely believed that all older persons develop dementia [2,3]. A nationally representative survey found that 77% of Americans aged 40 and older think that their cognition will decline [4]. According to the World Health Organization (WHO), the best way to measure cognitive and physical capacities in later life is to classify these domains as either showing or not showing decline [5,6]. Therefore, the corresponding WHO assessment tool does not allow for the possibility of improvement in these domains [5].

The following are among the reasons that aging is often viewed as a time of inevitable and universal decline. In aging science, there is a tendency to average patterns of older participants’ health outcomes over time, which can obfuscate subsets that show improvement [7]. Also, when the health of older persons shows improvement, it tends to be viewed as an exception to the general stigmatized category of older persons [3,8].

In contrast to the predominant trend of focusing on aging health as a time of decline, the current study considered whether a meaningful percentage of older persons would show health improvement when methods focused on whether this health improvement is experienced. It also considered for the first time whether positive age beliefs predicted this potential improvement. Most studies that have explored the determinants of aging health focused on factors that are enduring (e.g., genes), as well as negative and on the individual level (e.g., smoking), whereas the age beliefs on which we focused are modifiable, positive, and societal-based.

We expected to find this improvement would occur among a meaningful number of older persons and positive age beliefs would lead to improvement because, according to stereotype embodiment theory (SET) and its supporting research, individuals internalize both positive and negative age beliefs from environmental sources, such as social media, starting at a young age; in later life, when these beliefs become self-relevant, they can predict better or worse cognitive and physical health, respectively [3,9]. As individuals enter old age, according to SET, they are increasingly made aware of it by clues from their interpersonal and institutional contacts [3]. Consequentially, the age beliefs shift from only being applied to others to also being applied to oneself. This self-relevance magnifies their beliefs’ salience and, therefore, the associated health pattern [10].

The predicted pattern of improvement among those with positive age beliefs is also supported by our previous field study, which found that when older persons were exposed to a positive-age-belief intervention, their physical function continued to improve over the two-month follow-up time [10]. This was interpreted as illustrating a snowball effect such that the positive age stereotypes led to better physical functioning, which, in turn, led to more positive age stereotypes, which led to even greater physical function [3,10].

The expected linkage between age beliefs and health has been demonstrated by studies in at least 9 meta-analyses [11,12]. Specifically, the current study will build on three types of research relating to older persons: longitudinal studies that show positive age beliefs predict less decline in cognitive and physical function [13,14,15]; recovery studies that found, among older participants who had a physical disability or mild cognitive impairment at baseline, those who had assimilated more positive age beliefs were significantly more likely to regain functioning to achieve normal levels over time [16,17]; and positive-age- belief interventions that have benefited older persons’ memory and walking speed up to two months later [10,18].

In the current study, we built on previous age-stereotype research by examining whether improvement in functioning occurs in the community, outside of the laboratory, among older persons over time, including those who had all levels of functioning at baseline. We hypothesized that (1) a meaningful number of older persons will show improvement beyond their baseline levels in cognitive and/or physical functioning; and (2) positive age beliefs will predict this improvement over time. By utilizing a longitudinal nationally representative study, we were able to examine whether the older participants improved over a span of up to 12 years.

## 2. Materials and Methods

### 2.1. Sample

Participants were drawn from the Health and Retirement Study (HRS). It is supported by the National Institute of Aging and conducted by investigators at the Institute of Social Research at the University of Michigan. The HRS is based on biennial surveys of a nationally representative sample of Americans aged 50 and older [19].

Our study sample included all HRS participants for whom there were baseline measures of age beliefs and covariates, as well as at least one follow-up survey with measures of Telephone Interview for Cognitive Status (TICS) or walking speed [20,21]. The age-belief measure was first administered to a random half of the HRS sample in 2008, and administered to the other half in 2010 [22]. In the analyses, the participants’ first wave of age beliefs became the baseline. Whereas the HRS investigators administered TICS to all participants who were aged 50 and older, they only assessed walking speed in the participants who were 65 and older [23]. We followed participants for up to 12 years. The average follow-up period in the TICS cohort was 8.04 years (*SD* = 3.27), with a range of 2 to 12 years, and the average follow-up period in the walking-speed cohort was 8.54 years (*SD* = 2.86), with a range of 2 to 12 years. Most (76.43%) remained in the study for 10 years or more.

The final cohort of those in the cognitive analyses consisted of 11,314 participants, with an average baseline age of 68.12 (*SD* = 9.92), with ages ranging from 50 to 99 years. Most had a high-school education or greater (83%, with 23% having completed college) and were married (63%). The final cohort in the walking-speed analyses consisted of 4638 participants, with an average baseline age of 74.03 years (*SD* = 6.07), with ages ranging from 65.00 to 99.08 years. Most had a high-school education or greater (84%, with 24% having completed college) and were married (63%) (Table 1).

### 2.2. Measures

#### 2.2.1. Predictor: Positive Age Beliefs

**Figure 1:**
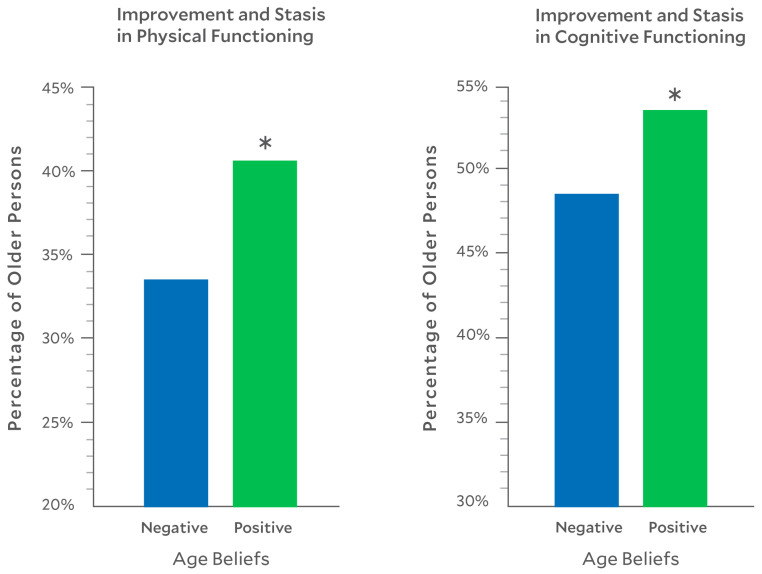
Positive age beliefs predict physical and cognitive improvement and stasis. Note: * *p* < 0.05. In the physical improvement and stasis group, 58% improved, and in the cognitive improvement and stasis group, 40% improved.

Age beliefs were assessed with the five-item Attitude Toward Aging subscale of the Philadelphia Geriatric Center Morale Scale (e.g., *The older I get*, *the more useless I feel* and *I am as happy now as I was when I was younger*) [24,25]. Potential responses range from *strongly disagree* to *strongly agree*. We reverse-scored responses of negative items so that total scores ranged from 5 to 30, with a higher score indicating more-positive age beliefs. The scale has good internal and external validity for older persons [17,24,25,26]. For Figure 1, we dichotomized participants at the age-belief median into groups based on their holding negative (≤15) or positive (>15) age beliefs.

#### 2.2.2. Outcome: Improvement in Health

##### Cognitive Improvement

Cognitive improvement was assessed with the 27-point version of TICS, a global measure of cognition that covers a range of cognitive domains, including short-term memory, delayed recall, and mathematical skills, and which has been validated with gold-standard clinical assessments and with other datasets that include similar-aged participants [21,27]. An additional advantage of the 27-point TICS version is that this was used to create the Langa–Weir normal cognition cut-point that allowed us to conduct a sensitivity analysis that repeated analyses with the subset of participants who had normal cognition at baseline [27]. To avoid practice effects, the HRS investigators created four non-overlapping TICS word lists to measure memory in different waves [28]. TICS is valid and reliable in assessing the cognitive function of older adults whose education is similar to our sample; it shows little evidence of ceiling and/or practice effects and has good sensitivity and specificity for identifying dementia [20,27,29].

To measure cognitive improvement, we subtracted participants’ baseline TICS score from the TICS score taken at their last follow-up wave, such that a larger number indicated an improvement in cognition. Thus, they received a score of 1 if their cognition improved (value above 0), and a score of 0 if their cognition stayed the same or declined (value at 0 or below).

##### Physical Improvement

Physical improvement was assessed by measuring walking speed, a global measure of physical function, which is often referred to as the “sixth vital sign” because it predicts lower rates of hospitalizations, disability, and mortality among older persons [30,31]. In HRS, participants were asked to walk at a “normal speed” and use any walking devices, including canes or walkers, that they find helpful. Researchers timed the participants’ walking for 8.21 feet or 2.5 m, and then calculated speed by dividing the distance (in meters) by the time recorded (in seconds) so that a larger number indicates faster speed [23]. Participants were asked to do this two times at baseline and at each of the follow-up waves. To assess maximum capability, we used the faster of the two times. At baseline, the average walking speed was 84.57 cm/s (*SD* = 23.86) with a range of 7.31 to 125 cm/s.

To measure physical improvement, we subtracted participants’ baseline walking speed from the walking speed taken at their last follow-up wave, such that a larger number indicated an improvement in walking speed. Thus, they received a score of 1 if their walking speed improved (value above 0), and a score of 0 if they stayed the same or walked slower (value at 0 or below).

#### 2.2.3. Covariates

We included covariates that have been found to be related to the predictor or the outcome [12,16,17]: age, sex, race and ethnicity (defined as White, Black or other ethic minority groups, which included American Indian, Alaska Native, Asian, and Native Hawaiian or other Pacific Islander), depressive symptoms (assessed with the validated 8-item Center for Epidemiological Studies Depression Scale, using the high-symptom cut-off score, associated with major depression, of 3 or greater) [32,33], sleep problems (assessed with the Jenkins Sleep Scale [34], which measures the frequency of sleep problems), education, marital status, cardiovascular disease/diabetes (assessed with reports that a health care provider diagnosed heart disease, hypertension, or diabetes), *APOE 4*, social isolation, and the number of years participated in HRS.

### 2.3. Statistical Analysis

To examine the first hypothesis, that a meaningful number of older persons will show improvement beyond the baseline level in cognitive and/or physical functioning, we calculated the percentage of the sample that showed improvement in each of these domains separately, from baseline to the last measurement up to 12 years later. Our operationalization of “a meaningful number of older persons” was based on a benchmark used to assess one of the few national targets for older persons set by the US Department of Health and Human Services: according to its publication, *Healthy People 2030*, the country should aim to increase distribution of preventive services to a meaningful number of at least 11.5% of older persons [35]. Thus, we will determine whether improvement occurs with 11.5% or more of the participants.

We examined the percentage of participants who showed improvement in cognition and/or walking speed. To do this, we measured the subset of HRS participants who had both cognitive and physical improvement scores. To make sure that we counted each participant only once in this total, we summed the percentages of those who showed improvement in cognition with those who showed improvement in walking speed, and then subtracted the percentage who showed improvement in both.

As stasis is another way that beliefs of inevitable decline are contradicted, in a secondary analysis, we studied instances of cognitive and physical functioning remaining consistent from baseline to the final assessments, up to 12 years later. Thus, we also examined the percentage of the sample that showed stasis or improvement, in one or both outcomes, by adding those who received the same score at baseline and in their last wave to the improvement category. Accordingly, this percentage included those with cognitive or physical scores greater than or equal to 0.

To examine the second hypothesis, that positive age beliefs will predict improvement in cognitive and physical functioning over time, we conducted logistic regression models with the positive-age-belief measure acting as the independent variable, both with and without covariates. Also, we repeated both sets of models with the outcome that added those showing stasis to the improvement group.

We also conducted a series of sensitivity analyses to increase our confidence in the robustness of the results. In a first set of sensitivity analyses, to be assured that the improvement in cognition and physical function was not just reflecting a regression to the mean, we repeated all of the analyses after removing the participants who showed a change in scores closest to 0, so that we limited the analysis to those who showed improvement described as substantial in the literature [36,37]. We repeated the cognition models with those who showed an increase of more than 1 point on the total TICS score, and compared them to those who showed a decline of more than 1 point on the total TICS score from baseline to last measurement recorded [36]. We also repeated the parallel walking-speed models, in this case, with the participants who showed an improvement in walking speed of greater than 5 cm/s, and compared them to those who showed a decline of more than 5 cm/s [37].

In a second sensitivity analysis, to examine whether the same pattern of findings addressing the two hypotheses was found with participants who have normal cognition and/or normal walking speed at baseline, we repeated the cognition models with those who met the definition of normal cognition by receiving a score of 12 or greater on the TICS [38] or who started with a normal baseline walking speed, defined as those who had a walking speed of greater than 60 cm/s or who took less than 4.2 s to walk 2.5 m, a usual cut-point distinguishing normal from slow-walking speed in a similar-aged population [39]. In a third sensitivity analysis, we repeated these positive-age-belief sensitivity models with the outcome that added those showing stasis to the improvement group.

## 3. Results

### 3.1. Hypothesis 1: Cognitive and Physical Improvement

As predicted by the first hypothesis, a meaningful number of older persons showed improvement beyond baseline level in cognitive and/ or physical functioning. Among participants with measurements of both domains, 45.15% showed improvement in cognition and/or walking speed from baseline to the last measurement up to 12 years later. By examining the domains separately, we found 31.88% improved their cognition, and 28.00% improved their walking speed. These percentages exceeded the *Healthy People 2030* target for a meaningful number of older persons of at least 11.5% by a significant margin.

Next, we expanded our category of those who defied the negative age belief of decline: participants who showed stasis were added. The percentages of participants who were stable or improved was 51.06% for cognition and 37.56% for walking speed (see Figure 1).

The correlation of participants who showed improvement in cognition and walking speed was r = 0.09. It was found that 44% of participants who showed improvement in cognitive function also showed improvement in walking speed.

It should be noted that in our study, when all participants were treated as a homogenous group, and their scores were averaged, as occurs in many studies, a decline was detected with TICS scores going down by 1.39 points and walking speed decreasing by 11.69 cm/s. However, when heterogeneity of the sample was considered, and improvement was given a focus, it became clear that a meaningful percentage of older persons improved with both measures.

To assess the robustness of our results related to improvement, two sets of sensitivity analyses were conducted. In the first set, to increase our confidence that a meaningful number of older persons showed improvement in cognition and/or physical function, we repeated the analyses with the more conservative definition requiring an improvement of more than 1 point on the TICS or an increase greater than 5 cm/s in walking speed. The predicted patterns remained: 22.50% showed improvement in cognition, and 26.71% showed improvement in walking speed, up to 12 years later.

Among the participants who showed improvement in cognition, most (71.98%) showed improvement of 2 points or more. Similarly, among those who showed improvement in walking speed, most (77.38%) showed improvement of 5 cm/s or more.

In the second set of sensitivity analyses, conducted with the subsample of those who had normal functioning at baseline, we found that a similar pattern of results emerged as in the full sample that included all levels of functioning. In the subsample categorized as normal at baseline, 27.74% showed improvement in cognition between baseline and the final assessment, up to 12 years later. In the parallel model, conducted with those who had normal walking-speed at baseline, 23.08% of this showed improvement in walking speed during this period. We also found that 47.64% showed stasis or improvement in cognition, and 32.37% showed stasis or improvement in walking speed.

### 3.2. Hypothesis 2: Health Improvement Predicted by Positive Age Beliefs

For the primary model addressing the second hypothesis, positive age beliefs predicted improvement in cognitive health, both with (OR = 1.04, 95% CI 1.00–1.08, *p*= 0.049) and without covariates (OR = 1.06, 95% CI 1.02–1.10, *p* = 0.002), as well as in walking speed, both with (OR = 1.09, 95% 1.02–1.17, *p* = 0.018) and without covariates (OR = 1.11, 95% CI 1.04–1.18, *p* = 0.001). The same pattern was found when we repeated this model with the stasis group included (See Figure 1).

To ensure the robustness of our results with positive age beliefs as a predictor of improvement, we conducted two sets of sensitivity analyses. The first set examined whether the significant pattern of results remained when using a more conservative definition of cognitive improvement, by requiring it to be greater than 1 point. Positive age beliefs still predicted cognitive improvement, both with (OR = 1.05, 95% CI 1.00–1.11, *p* = 0.03) and without covariates (OR = 1.07, 95% CI 1.03–1.12, *p* = 0.0008). Similarly, in the model with greater than 5 cm/s walking-speed improvement as the outcome, positive age beliefs predicted it, both with (OR = 1.09, 95% CI 1.01–1.18, *p* = 0.023) and without covariates (OR = 1.12, 95% CI 1.04–1.20, *p* = 0.001).

In the second set of sensitivity analyses, conducted with the subset of participants classified as having normal cognition at baseline, positive age beliefs predicted cognitive improvement, both with (OR = 1.06, 95% CI 1.01–1.10, *p* = 0.02) and without covariates (OR = 1.12, 95% CI 1.08–1.16, *p* < 0.001). In the parallel model, conducted with those who had normal walking-speed at baseline, it was also found that positive age beliefs predicted improvement, both with (OR = 1.14, 95% CI 1.05–1.24, *p* = 0.0016) and without covariates (OR = 1.19, 95% CI 1.10–1.27, *p* < 0.001). The same pattern was found when this model was repeated with the stasis group included, both with (OR = 1.08, 95% CI 1.04–1.13, *p* = 0.002) and without covariates (OR = 1.14, 95% CI 1.10–1.18, *p* < 0.001).

In the third set of sensitivity analyses, when we combined the two earlier sensitivity analyses by using the conservative-improvement definitions with the subset of participants without deficits at baseline, the same patterns emerged. Positive age beliefs still predicted cognitive improvement, with (OR = 1.08, 95% CI 1.02–1.15, *p* = 0.006) and without covariates (OR = 1.16, 95% CI 1.10–1.22, *p* < 0.001), as well as physical-function improvement, with (OR = 1.17, 95% CI 1.07–1.29, *p* = 0.0009) and without covariates (OR = 1.22, 95% CI 1.20–1.32, *p* < 0.001).

## 4. Discussion

The current study demonstrated that the predominant narrative of aging as a time of inevitable and universal decline needs to be reconsidered. We discovered that 45.15% of the participants, who were 65 years and older, showed improvement in cognitive and/or physical function over a period of up to 12 years from baseline. If this finding was extrapolated to the entire US population, it would suggest that more than 26 million older persons are experiencing an improvement in functioning.

In addition, the current study demonstrated for the first time that participants who had assimilated more-positive age beliefs were more likely to show improvement in both cognitive and physical function. The robustness of these results was supported by several sensitivity analyses, including one that showed the significant pattern of health improvement remained when we limited the analyses to the subset with normal functioning at baseline. This suggests that the improvement, and its prediction by positive age beliefs, is not only due to participants with deficits recovering to a normal level of functioning, but also occurred for those who started at normal levels of functioning. Thus, it seems this improvement pattern is picking up on a cognitive and physical reserve that is available to the general population of older persons.

Our previous research suggests a possible explanation for the way age beliefs were able to exert their influence. We had found that the brain, which governs much of health, is susceptible to the influence of age beliefs. Specifically, in their negative form, these beliefs were associated with biomarkers of Alzheimer’s disease (i.e., accumulation of plaques and tangles, together with lower hippocampal volume) [40]. Future research should determine whether the opposite type of age beliefs, the positive ones, have the opposite association with brain activity by increasing the rate at which the neurons generate new connections in later life, and whether the positive age beliefs may contribute to the later-life regeneration of satellite cells in muscles.

Findings of the current study suggest there are concepts of aging science that could benefit from revisions. Rather than limiting biomarkers of aging to indicators of decline, there could be biomarkers of improvement and stability; the operationalization of geroscience could be expanded beyond predicting patterns of decline to also predicting improvement and stability in aging health; and the widespread use of the term “accelerated aging” could be expanded beyond its current definition, which equates aging with decline, to include patterns of improvement and stability.

Most 66% of the participants only showed improvement in one domain: cognition or walking speed. This contrasts with the widespread assumption that cognitive and physical health patterns exist in tandem in later life [35].

It is unlikely that the results of the current study were due to a selection effect, because the modeling did not allow attrition. That is, all participants had baseline and final follow-up assessments. We included all participants, regardless of how long they were in the study. Also, the length of time individuals remained in the study acted as a covariate in all the multivariate models. Further, most (76.43%) participants were in the study for 10 years or more. In all models, we adjusted for the factors that distinguished those who remained in the study for 10 years or more from those who remained in the study for shorter times.

The current study was limited by the HRS not including measures of muscle and brain neuron plasticity, which could help identify the mechanism of improvement. In future studies, it would be helpful to examine improvement patterns of additional types of cognition, such as spatial memory. In addition, although our participants were drawn from a nationally representative sample, it would be useful to examine patterns of improvement in additional cohorts that have a greater representation of different ethnic minority groups.

Our study has a number of strengths. We focused on an aspect of aging health that is rarely examined or acknowledged, improvement, and we studied this with what are considered gold-standard measures, in a nationally representative sample [21,30]. The cognitive and physical outcomes were based on performance rather than self-report, and these outcomes have been found to contribute to older persons’ quality of life [21,29,30,37]. We examined a predictor of improvement, positive age beliefs, that can be modified on both an individual and a societal level [3,10].

By demonstrating that health improvement occurred in a meaningful percentage of our sample, we debunked the age belief that later life is a time of inevitable and universal decline. In doing so, a basis is provided for raising some older persons’ expectations about their health, which might increase their self-efficacy, which could lead to greater engagement in health behaviors (that otherwise suffer from fatalism), which could contribute to further health improvement.

Awareness of our improvement findings might also influence those with whom older persons interact. For instance, it might overcome the unwillingness of some healthcare providers to offer older persons preventive and rehabilitation services, due to the assumption that they are unlikely to get better [3,41]

It would be accurate and beneficial to expand the prevailing definition of aging so that it includes the possibility of health improvement. Insofar as we found that positive age beliefs contributed to this outcome, it is imperative that researchers and policymakers seek and deploy ways to amplify them.

## Figures and Tables

**Table 1:** Cognitive-improvement and physical-improvement samples’ baseline information.

Cohort	Variable	Level	Frequency	Mean (*SD*) or %
Cognitive Improvement Sample	Age (years)		11,314	68.12 (9.92)

	Sleep problems (Jenkins Scale)		11,314	2.40 (0.60)

	Feel isolated		11,314	1.39 (0.61)

	TICS score		11,314	15.70 (4.14)

	Years in study		11,314	8.04 (3.27)

	Sex	Female	6683	59.07
		Male	4631	40.93
	Married	Yes	7125	62.98
		No	4189	37.03
	Race	Black	1541	13.62
		White	9165	81.01
		Other Ethnic Minority Groups	608	5.38
	High school or greater education	Yes	9446	83.49
		No	1868	16.51
	Depressive symptoms	Yes	1447	12.79
		No	9867	87.21
	Diabetes, heart disease, or hypertension	Yes	7850	69.38
		No	3464	30.61
Physical Improvement Sample	Age (years)		4638	74.03 (6.07)

	Sleep problems (Jenkins Scale)		4638	2.46 (0.58)

	Feel isolated		4638	1.31 (0.54)

	Walking speed		4638	84.57 (23.86)

	Years in study		4638	8.54 (2.86)

	Sex	Female	2708	58.39
		Male	1930	41.61
	Married	Yes	2941	63.41
		No	1697	36.59
	Race	Black	433	9.34
		White	4074	87.84
		Other Ethnic Minority Groups	131	2.82
	High school or greater education	Yes	3911	84.33
		No	727	15.67
	Depressive symptoms	Yes	408	8.80
		No	4230	91.20
	Diabetes, heart disease, or hypertension	Yes	3465	74.71
		No	1173	25.29

Note: Walking Speed was measured in centimeters/s. Depressive symptoms were measured with the 8-item Center for Epidemiologic Studies Depression Scale, scored from 0 to 8, with the validated high symptom cut-off score of ≥3.

## Data Availability

The HRS data documentation and dataset are available at: https://hrs.isr.umich.edu/about.
